# Supplementary material for: SLAV-Sim: A Framework for Self-Learning Autonomous Vehicle Simulation
Source: Sensors (Basel). 2023 Oct 23;23(20):8649. doi: 10.3390/s23208649 (PMC10611232; doi:10.3390/s23208649)
Supplement: Supplementary file 1 [file sensors-23-08649-s001.zip › sensors-2580736-supplementary.pdf]

1. INTRODUCTION

Welcome to the supplementary material accompanying our research paper on SLAV-Sim, a lightweight simulator designed to train the behaviour of self-learning autonomous vehicles. In addition to providing further information on the simulator's features, design principles, and contributions, this section also presents additional results that complement the main paper, enhancing the understanding of SLAV-Sim's capabilities and performance.

2. SCENARIOS AND THEIR BEHAVIOURS

The environment is split into different scenarios. Each scenario has a different road layout that represents a different part of driving. Each of these scenarios will have its own environment to allow the agent to train for certain aspects of driving in separate spaces. This also allows for comparisons between each algorithm when controlling the vehicle in different scenarios. The scenarios and their behaviours are demonstrated in Table S1.

Table S1. Table of scenarios

Name of Scenario	Behaviours required
Basic Driving	This scenario will train the agent to travel alone on a two-way road and tests the agents' lane keeping, cornering and smooth driving ability. This scenario monitors all aspects of basic driving. This layout was built to be simple to observe the basic driving ability needed on general roads.
Speeding	This scenario has a very low speed limit, which the agent must obey. Although all scenarios have speed limits, the speeding scenario has long straights and a very low speed limit. This means that the agent can speed up easily if it does not reduce throttle input. The long straights are used to force the agent to slow down manually and not for environmental reasons, such as corners. This will test the agent's ability to maintain a constant speed as close to the speed limit as possible.
Traffic	The traffic scenario is the same as the basic driving scenario. However, the traffic scenario has other self-learning vehicles populating the roads at the same time. This will test the agent's response to other road users and test behaviours like keeping distance from the vehicle in front and avoiding accidents.
Crossings	This scenario places 4 crossings on the basic loop. These crossings change from red to green at random times. The vehicle must wait for the light to be green before passing through the crossing. This checks an agent's ability to judge distance, as the agent will need to stop in a specific spot in front of the lights to get a reward.
Roundabout	In this scenario, the agent must navigate a roundabout and egress at the correct exit. The correct exit changes each time the agent approaches the roundabout. This will test an agent's ability to corner around a central point as well as an agent's ability to exit a roundabout correctly.

Table S1. *Cont.*

Name of Scenario	Behaviours required
4-Way Lights	This scenario is a set of lights at a crossroads. Each time the agent approaches, an exit is chosen. The agent must egress at the correct exit. This scenario has a very narrow central point that the agent must navigate. This tests the agent's ability to negotiate small spaces as well as judge distance in a small space. This tests an agent's pre-planning, as it will need to slow down before the traffic light in order to make the tight turn.
Motorway	The motorway scenario has a high-speed limit, and the agent must learn to approach this limit and control the vehicle, keeping in lane at high speeds. This layout was chosen because the high speed will accentuate the inputs made by the agent.

3. ADDITIONAL ALGORITHM RESULTS AND DISCUSSION

This section contains some additional graphs of the results, which include the comparison of four algorithms on other scenarios and the observation results on other scenarios. PPO was the most performant algorithm, as it gained a higher cumulative reward than the other agents in 5 out of 7 scenarios. The behaviour generated by this algorithm was also in line with the expected driving behaviour. Although the PPO algorithm crashed more frequently in the episode-length experiments, it drove closer to the speed limit than the other algorithms.

All algorithms get a reward for driving close to the speed limit. This reward increases the closer the agent is to the speed limit. PPO used this reward to outperform the other algorithms. In addition to getting a larger forward reward than the other algorithms, the PPO agent would encounter more rewards in the same time frame as the other algorithms by driving faster. This increased speed, however, did often lead to the agent crashing more frequently than the other algorithms. This high speed is also why PPO outperformed in the crossing scenario, as this agent was driving more quickly and failed to stop at the crossings. This was because the algorithm placed a high value on the forward reward. Once the reward for waiting at the crossing lights was discovered, it began to get a higher per-episode reward than SAC. This shows that the algorithm was stuck in a local optimum for the first 8 million steps of the crossing scenario but showed the expected behaviour when the wait reward was discovered.

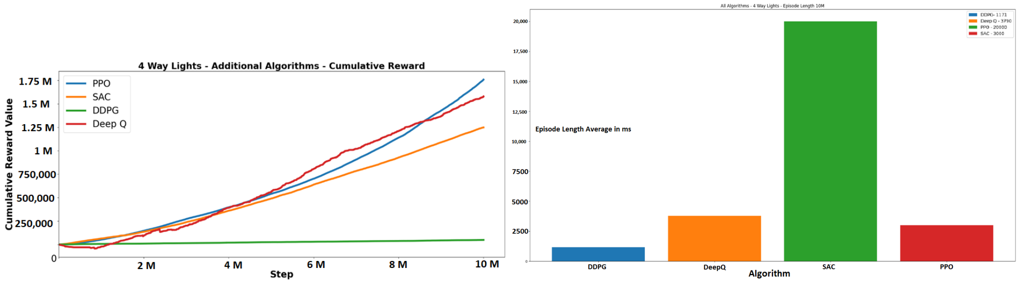

Figure S1. Cumulative reward and episode length for the 4-way lights scenario

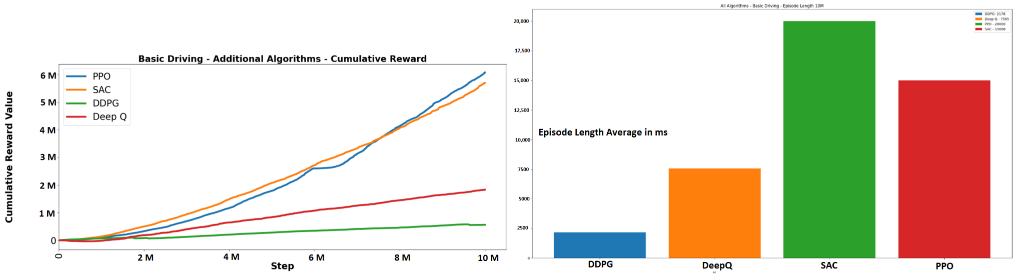

Figure S2. Cumulative reward and episode length for the Basic Driving Scenario

The SAC agent did not perform as well as the PPO agent when observing only the cumulative reward. This is because the SAC agent travelled slower than the speed limit and the PPO agent This slow speed meant that the agent was able to control the car better and traverse the environment more easily, therefore crashing less. This is an issue, however, because by driving more slowly, this agent missed out on gaining a substantial forward reward. By travelling more slowly, the SAC linear agent also travelled less distance in the allotted testing time than the PPO agent. This means that the agent covered less distance, meaning it had less opportunity to crash, which explains the low number of crashes during testing but also the lower cumulative reward. As SAC travelled slower than the PPO algorithm, it missed out on getting as much experience because it was in fewer unique states than its PPO counterpart. SAC was the most dominant algorithm as it showed desired driving behaviour earlier on in the training in 5/7 scenarios compared to the PPO. This suggests that the SAC agent was more sample-efficient.

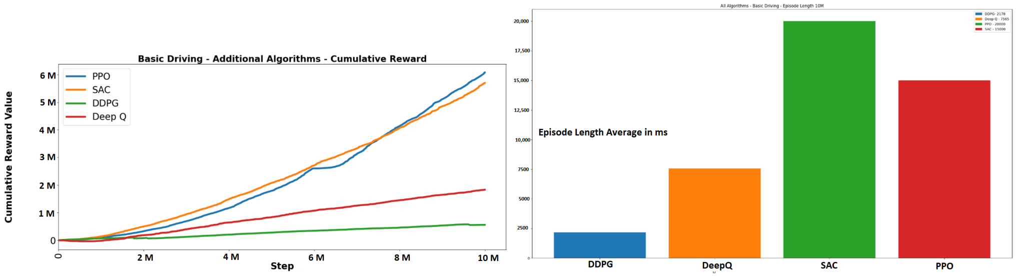

Figure S3. Cumulative reward and episode length for the Basic Driving Scenario

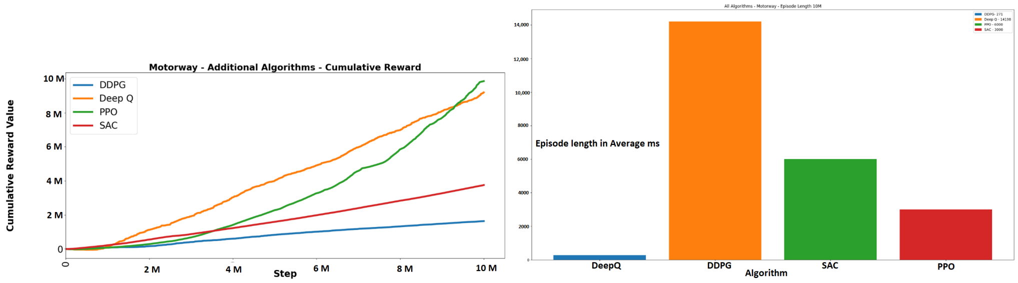

Figure S4. Cumulative reward and episode length for the Motorway Scenario

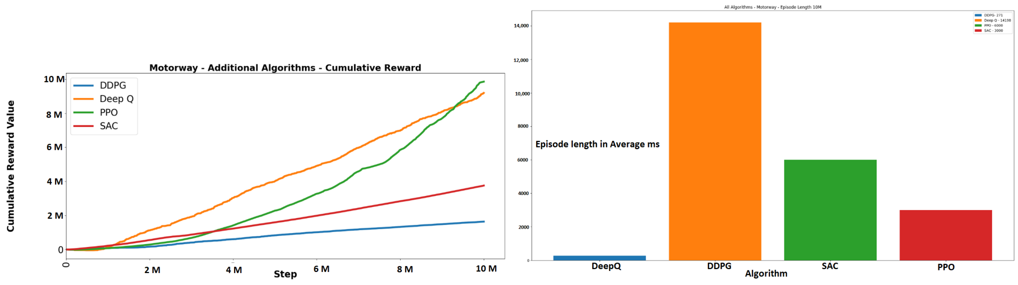

Figure S5. Cumulative reward and episode length for the Motorway Scenario

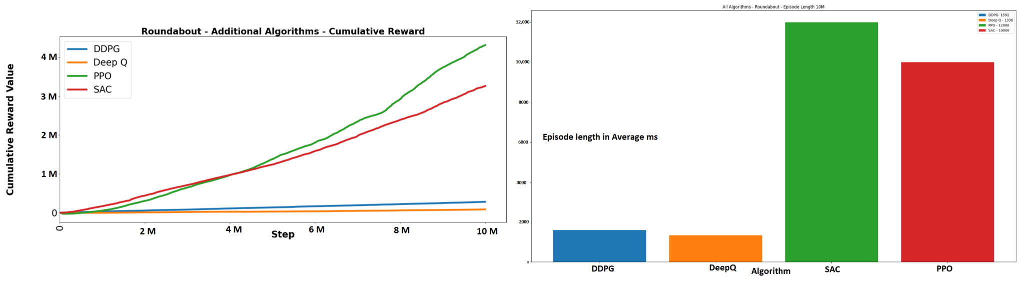

Figure S6. Cumulative reward and episode length for the Roundabout Scenario

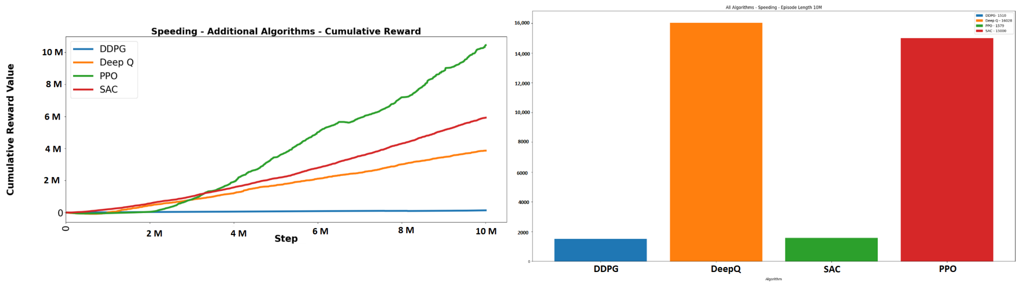

Figure S7. Cumulative reward and episode length for the Speeding Scenario

Deep Q often showed desirable behaviour; however, it did not achieve rewards as high as SAC or PPO. This algorithm often got a negative reward at the start for longer than the other algorithms and did not improve as rapidly. The Deep Q agent typically had longer episode lengths than the SAC and PPO agents. Deep Q got a lower score than SAC and PPO because this agent usually travels very slowly, which explains the long episode times. Therefore, the Deep Q agent missed out on possible rewards similar to those of the SAC agent. The Deep Q consistently showed the desired behaviour but did not perform as well as SAC or PPO due to its low speed.

4. INCREASED NETWORK SIZE RESULTS AND DISCUSSION

When using raycasts to observe the environment, there are a total of 12 observations that are made. 10 of these are raycasts, while the others consider the vehicle's speed and rotation. This small number of observations allowed for a basic algorithm setup that does not have many layers or hidden units; this saves on computation time and allows this benchmark to be run on low-end hardware for repeatability of results. Changing the observation method to a camera with a 20x20 resolution means that there are a total of 400 pixel observations made. As the observation complexity has increased, the algorithmic complexity must also increase. When using this kind of observation method, the algorithm size will now be doubled to have 256 hidden units and 5 layers. These new hyperparameters improved the performance of the algorithm overall but also

increased the computation time significantly. Comparing the camera results with the previous agents would not allow for a fair comparison because their network is much smaller, so a new raycast agent with the same network size has also been benchmarked. This new agent will be used to compare the two observation methods. All results in this section will use the PPO algorithm, as it consistently outperformed the other algorithms in the previous two experiments.

The data in Figure S8 shows that each observation method performed better in different scenarios. The raycast observation method performed better on 5/7 scenarios, most notably roundabouts, traffic lights, and 4-way lights. The camera performed better than the raycast in the basic driving and motorway scenarios. The raycast agent typically took less time to collect rewards at the start of training, gathering more rewards than the camera agent by the 2 million-step mark on 5/7 scenarios. This is because passing fewer observations allowed the algorithm to produce the driving behaviour quicker because of the reduced number of data points. By looking at the scenarios that each observation method performed best in, it can be concluded that the lack of accurate distance information was an issue for the camera agent. On top of this, the camera only observed the road in front of the vehicle. This was an issue in scenarios such as the traffic lights and the crossings because the camera was unable to calculate the distance to the traffic light accurately. This led to the camera agent crashing more frequently in scenarios that required accurate distance information and, therefore, a lower reward as a result. The camera sensor does have advantages, which are shown by the basic driving and motorway scenarios where the camera agent outperformed the raycast agent. This performance improvement was because the camera agent had more information about the upcoming road, as the camera observation method focuses entirely on the forward direction and passes more information to the agent than the three raycasts present at the front of the raycast agent. The raycast information, while enough to produce desired driving behaviour in these scenarios, did not give the agent as much information as the camera. The camera has a 20x20 resolution, all of which is pointed in front of the vehicle. This gave the camera agent more information about the upcoming road than the raycast agent. Therefore, the camera agent performed better and crashed less frequently.

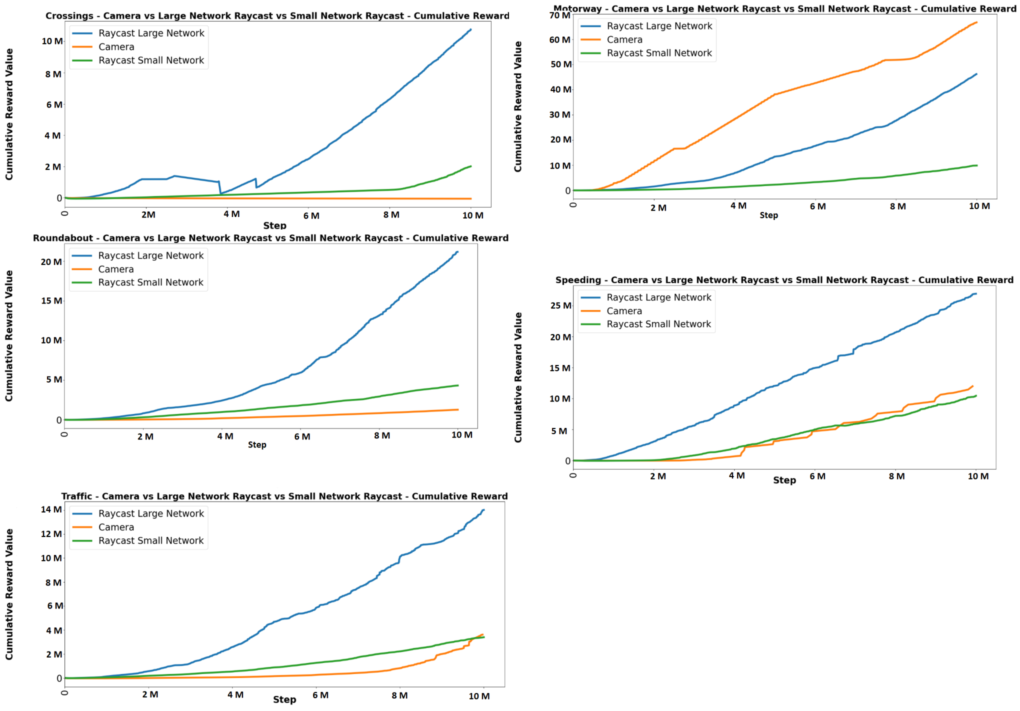

Figure S8. Camera vs large network raycast vs small network raycast cumulative reward comparison for different scenarios
