# Supplementary figures and images for: SLAV-Sim: A Framework for Self-Learning Autonomous Vehicle Simulation
Source: Sensors (Basel). 2023 Oct 23;23(20):8649. doi: 10.3390/s23208649 (PMC10611232; doi:10.3390/s23208649)

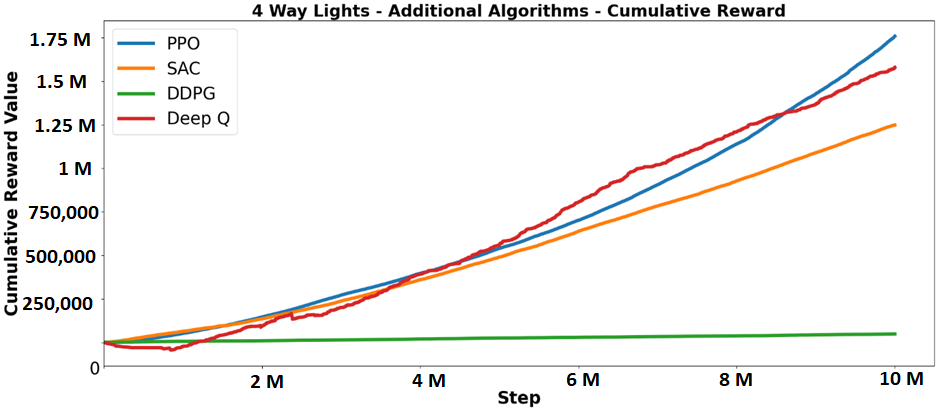

Supplement: Supplementary file 1 [file sensors-23-08649-s001.zip › images/4_way_lights_CL.png]

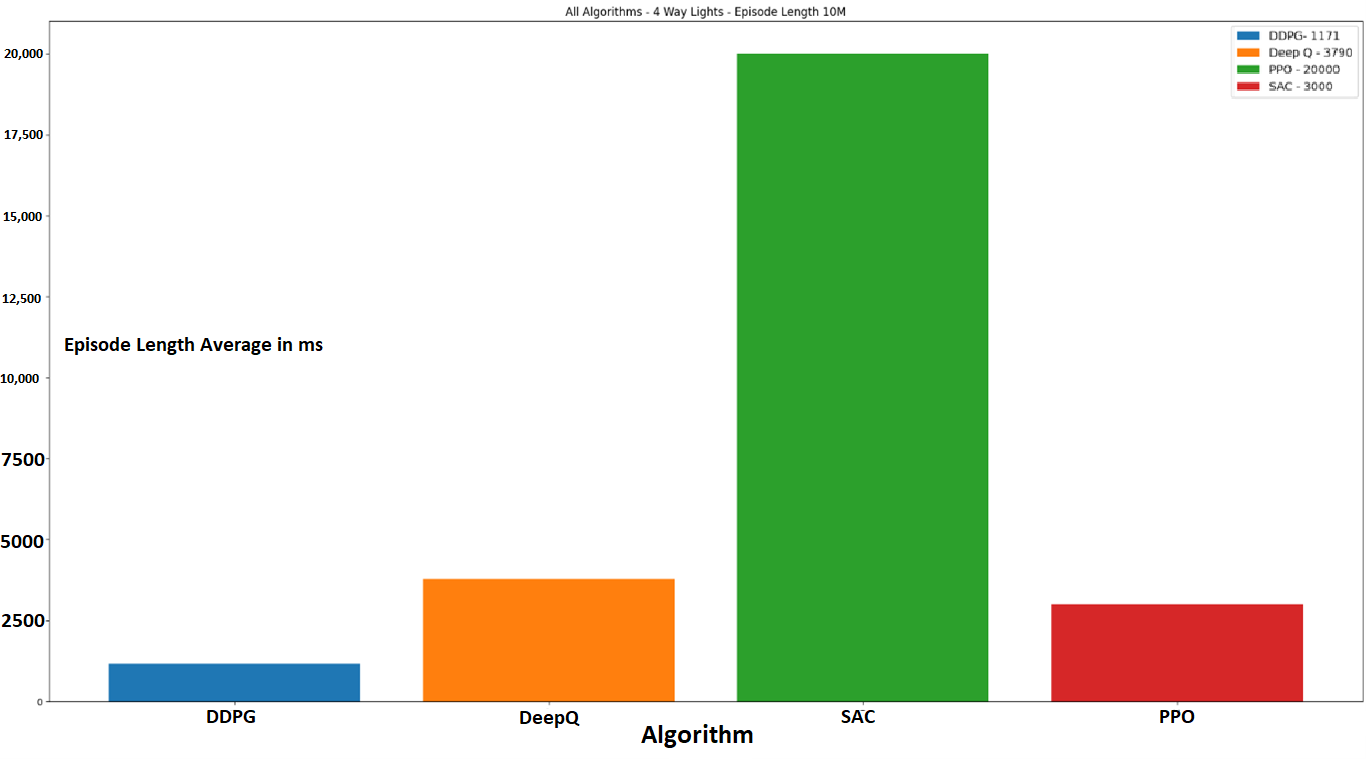

Supplement: Supplementary file 1 [file sensors-23-08649-s001.zip › images/4_way_lights_EL.png]

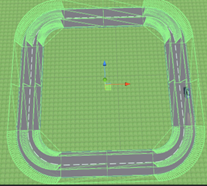

Supplement: Supplementary file 1 [file sensors-23-08649-s001.zip › images/basic_driving.png]

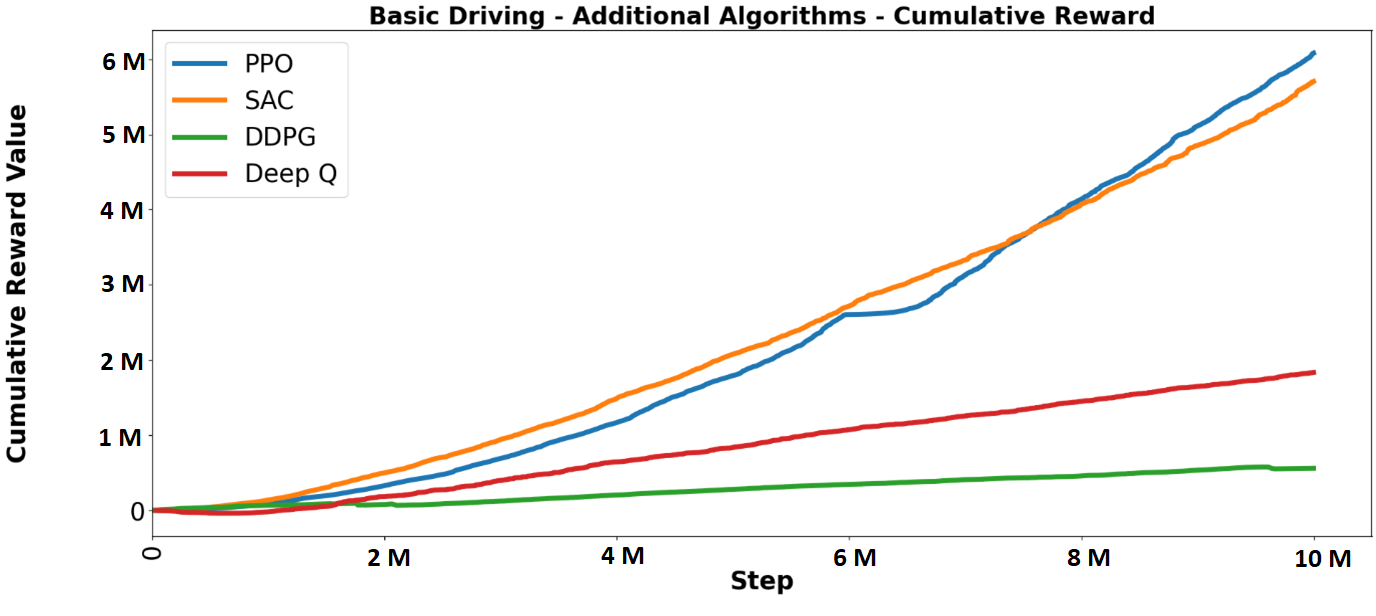

Supplement: Supplementary file 1 [file sensors-23-08649-s001.zip › images/Basic_Driving_CL.png]

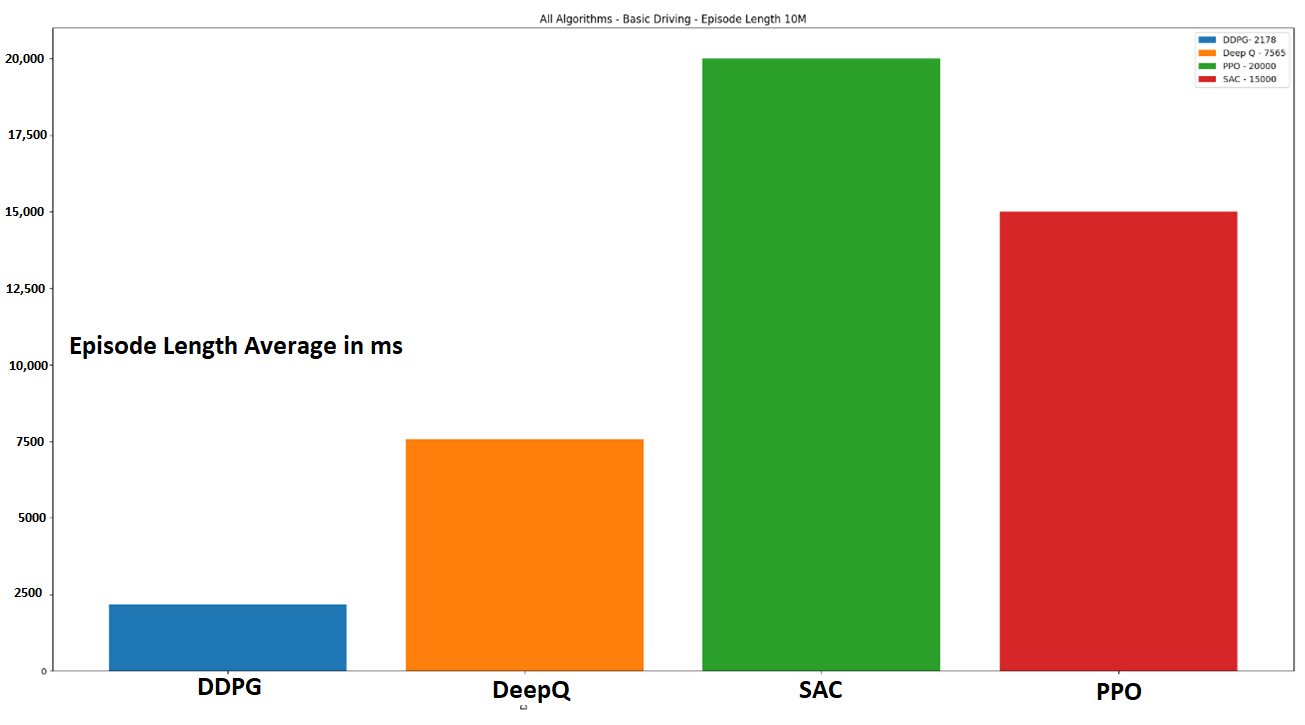

Supplement: Supplementary file 1 [file sensors-23-08649-s001.zip › images/Basic_Driving_EL.png]

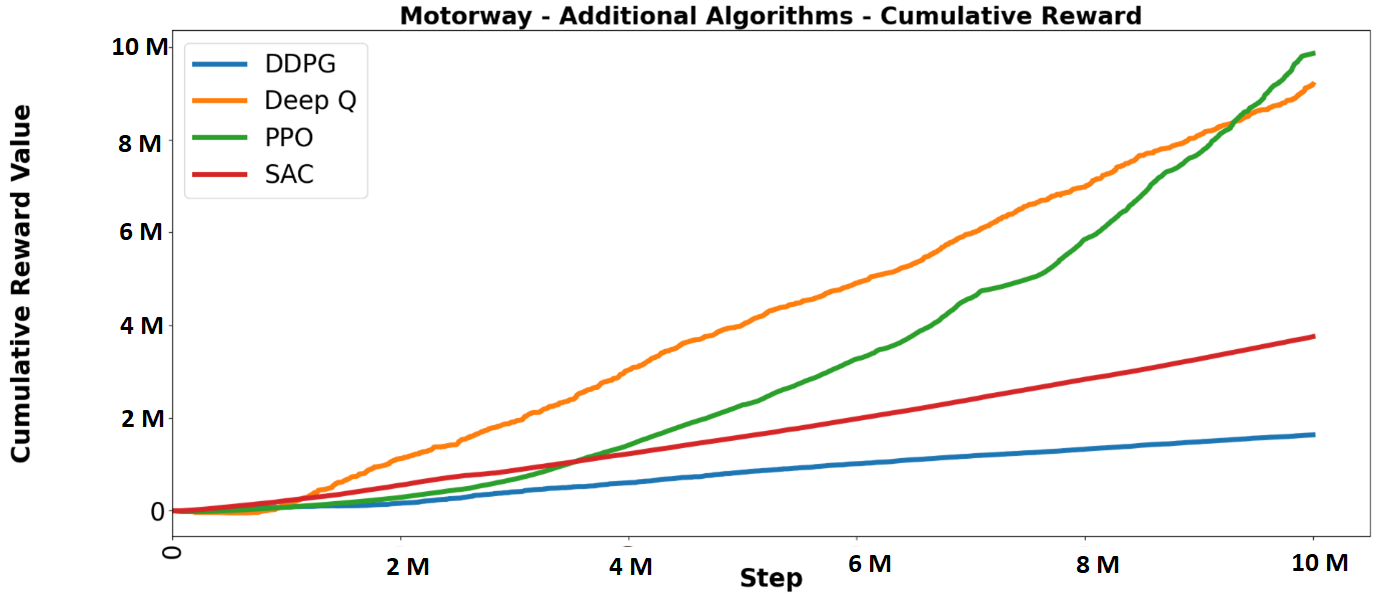

Supplement: Supplementary file 1 [file sensors-23-08649-s001.zip › images/Motorway_CR.png]

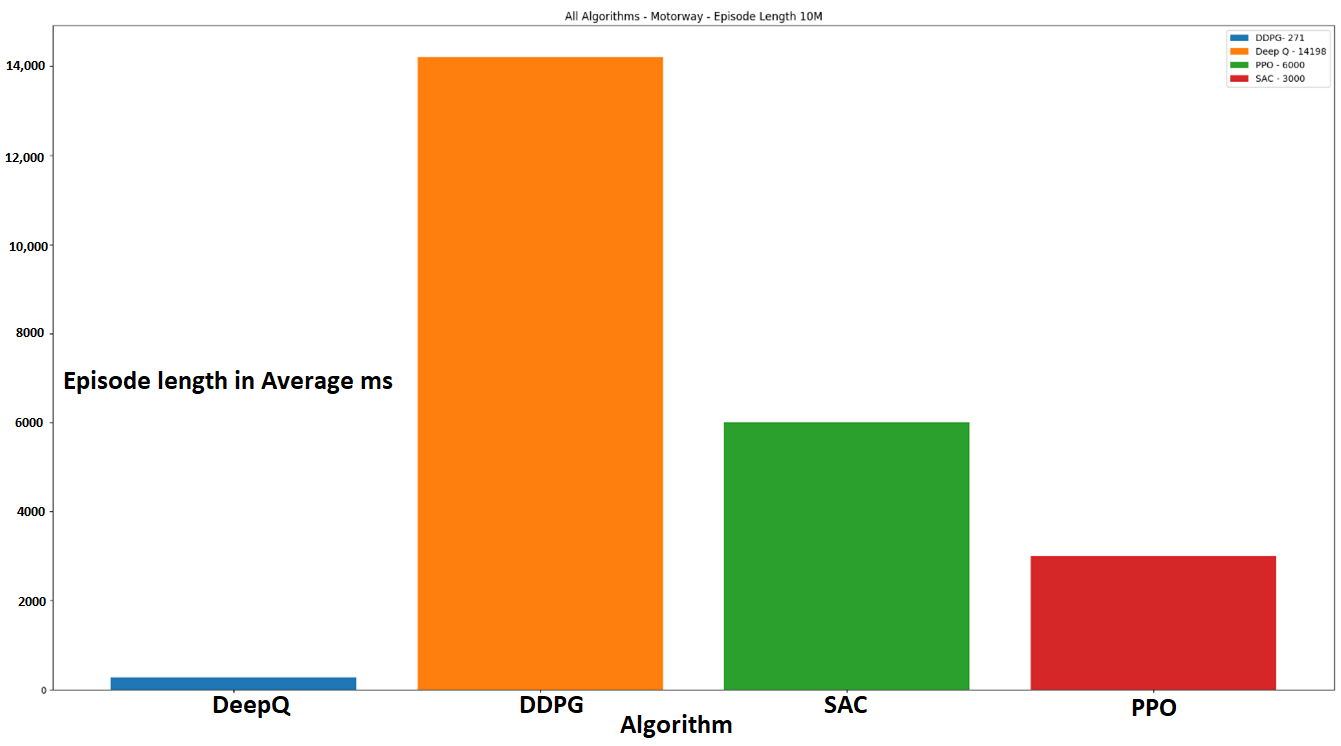

Supplement: Supplementary file 1 [file sensors-23-08649-s001.zip › images/Motorway_EL.png]

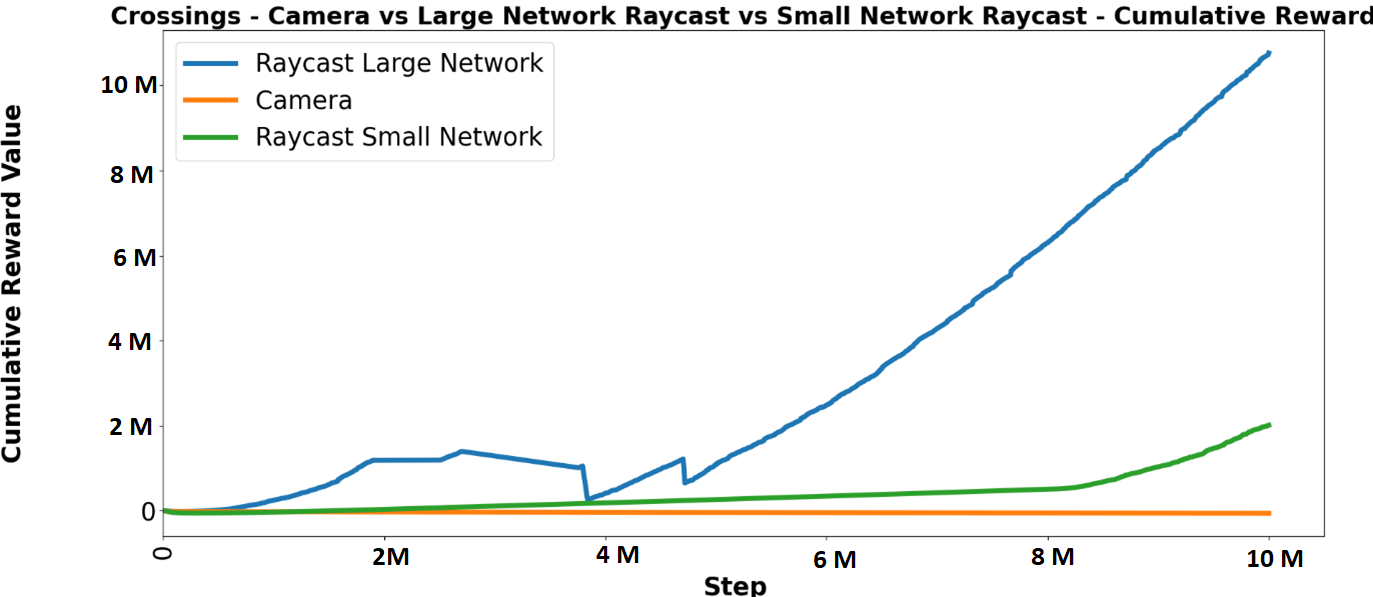

Supplement: Supplementary file 1 [file sensors-23-08649-s001.zip › images/Obs_Crossings.png]

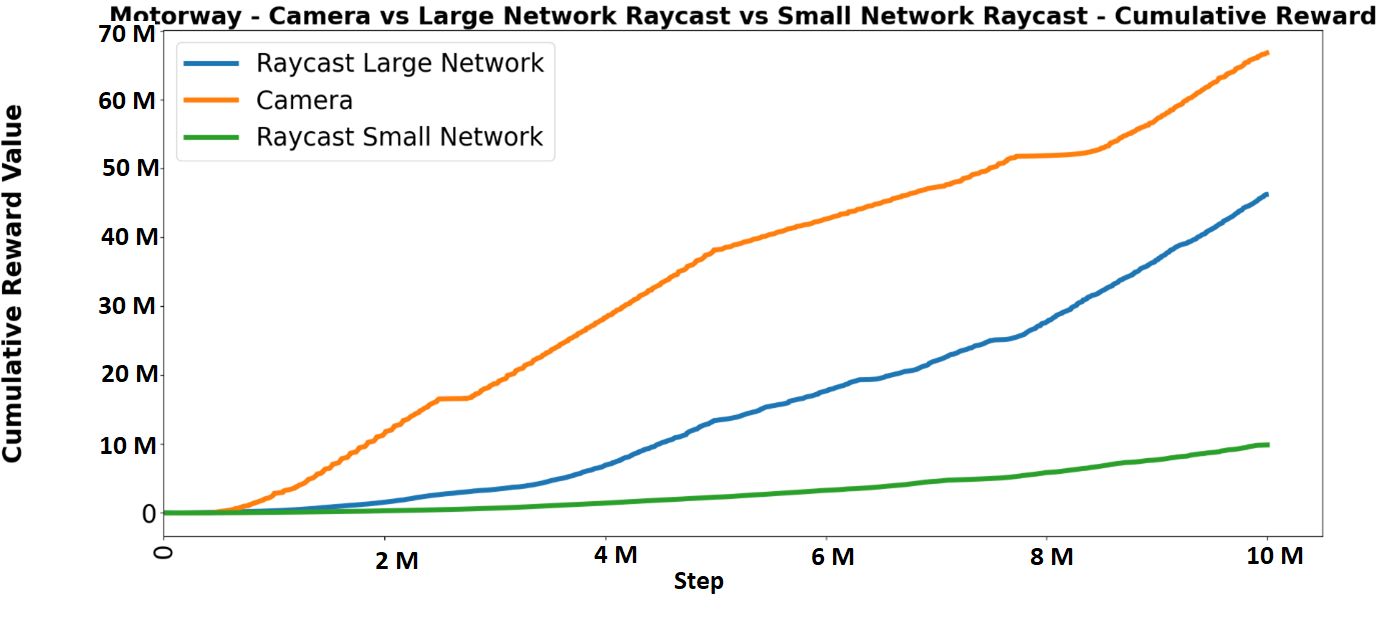

Supplement: Supplementary file 1 [file sensors-23-08649-s001.zip › images/Obs_Motorway.png]

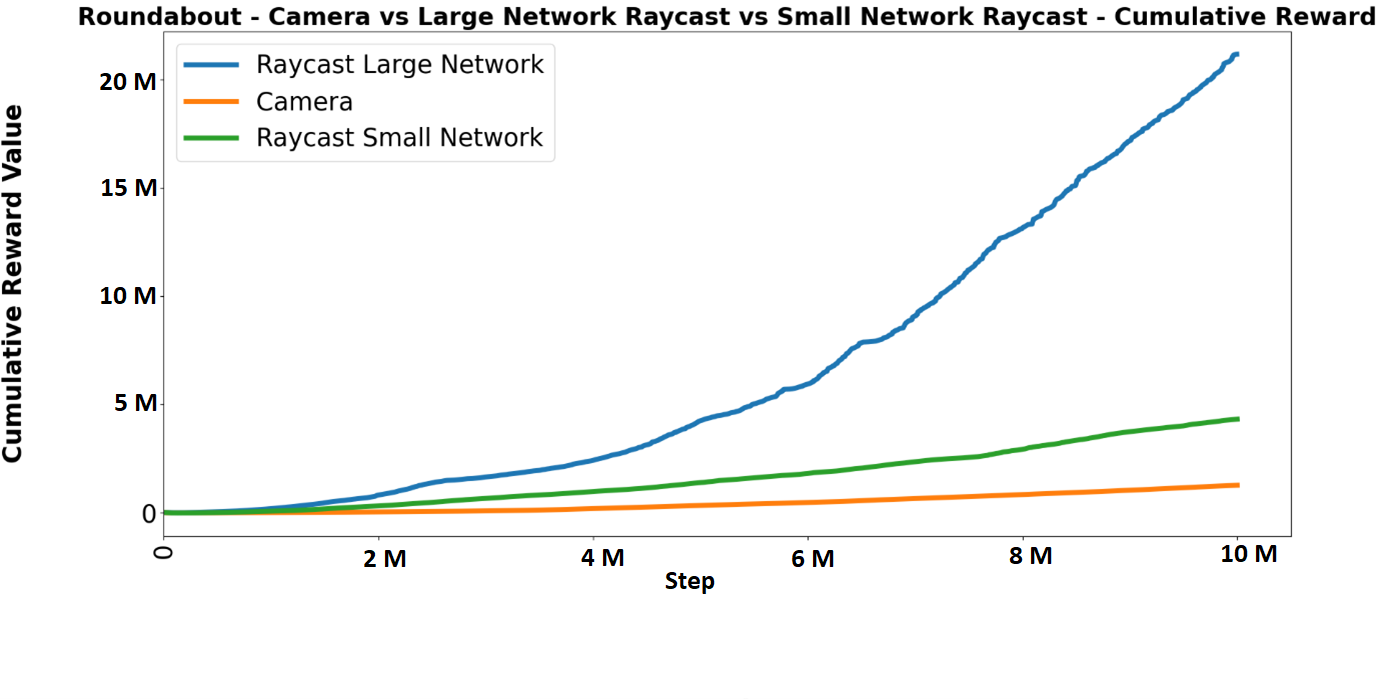

Supplement: Supplementary file 1 [file sensors-23-08649-s001.zip › images/Obs_Roundabout.png]

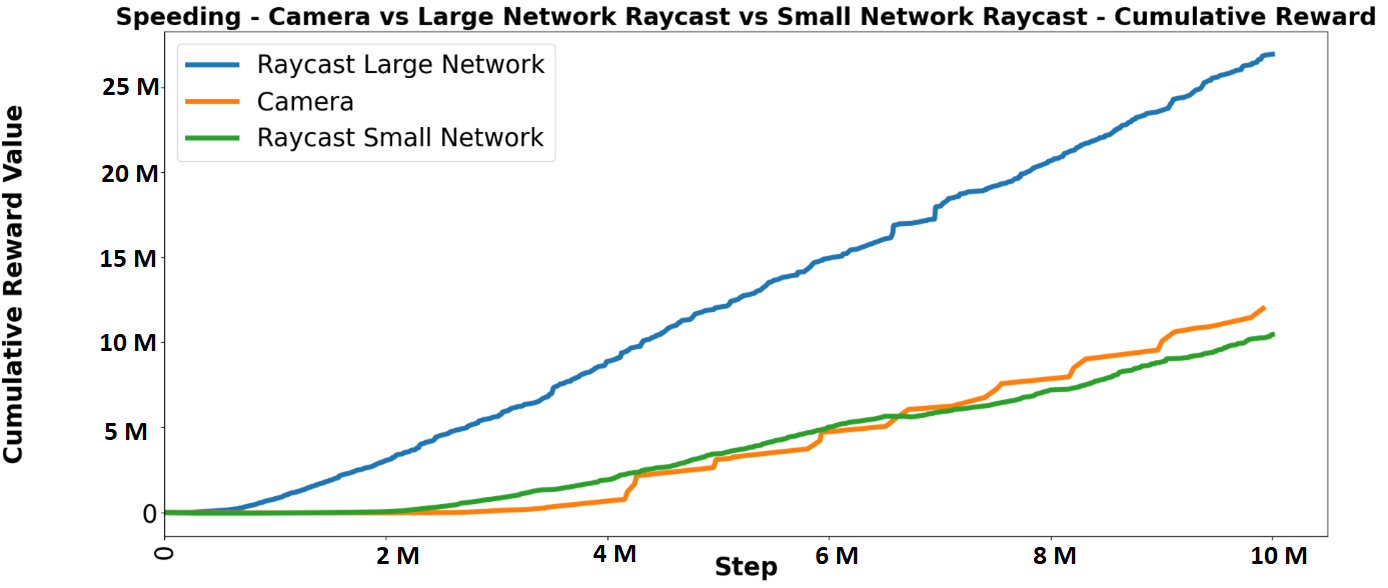

Supplement: Supplementary file 1 [file sensors-23-08649-s001.zip › images/Obs_Speeding.png]

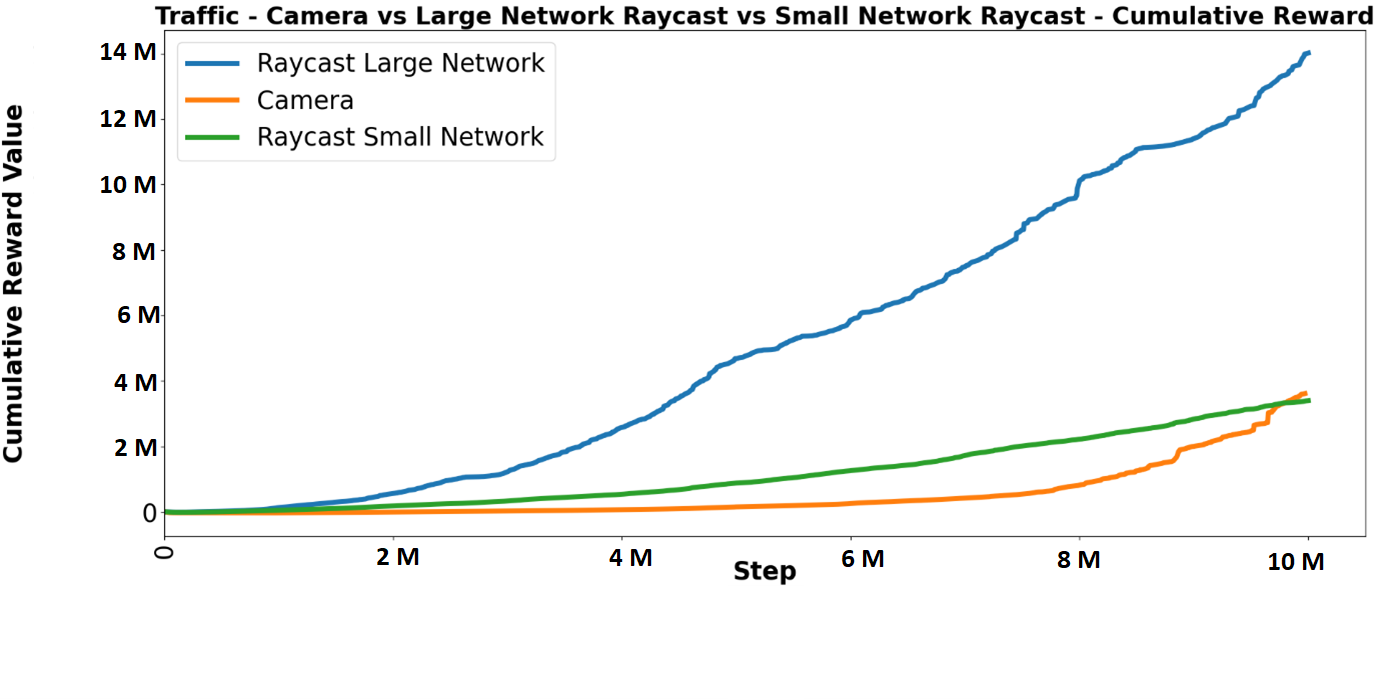

Supplement: Supplementary file 1 [file sensors-23-08649-s001.zip › images/Obs_Traffic.png]

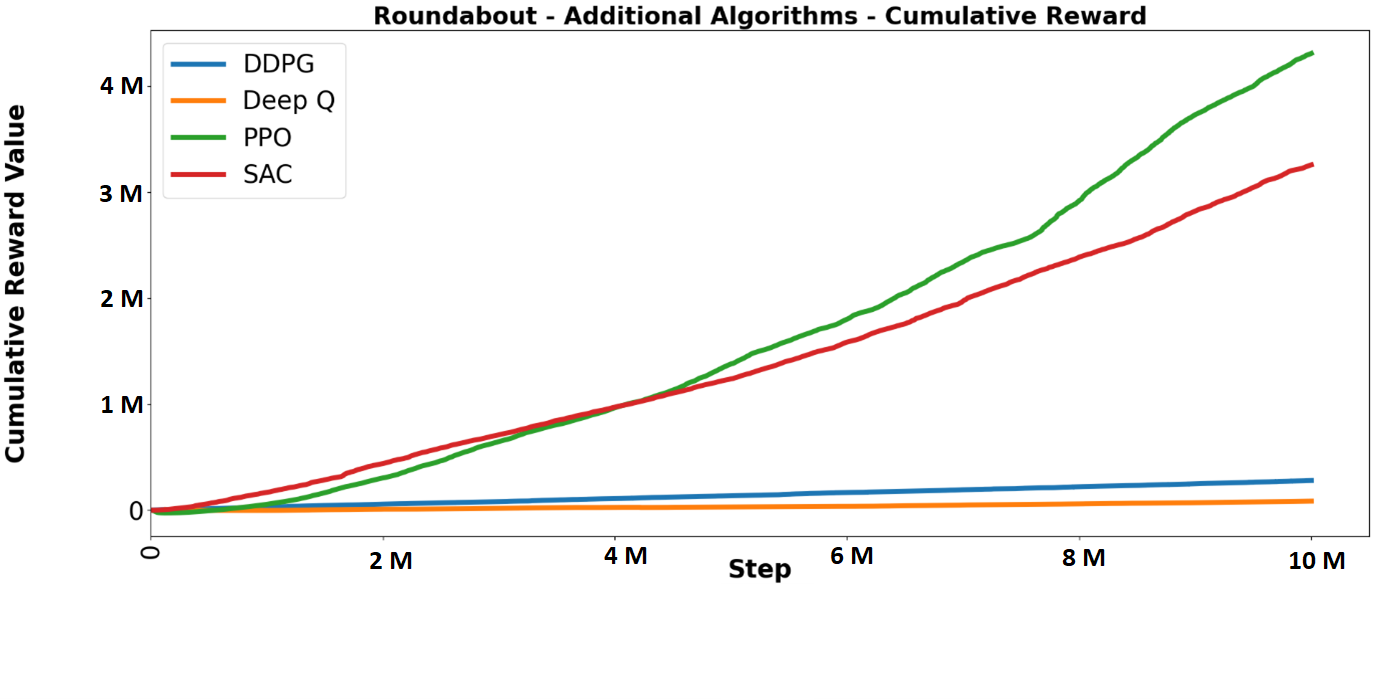

Supplement: Supplementary file 1 [file sensors-23-08649-s001.zip › images/Roundabout_CL.png]

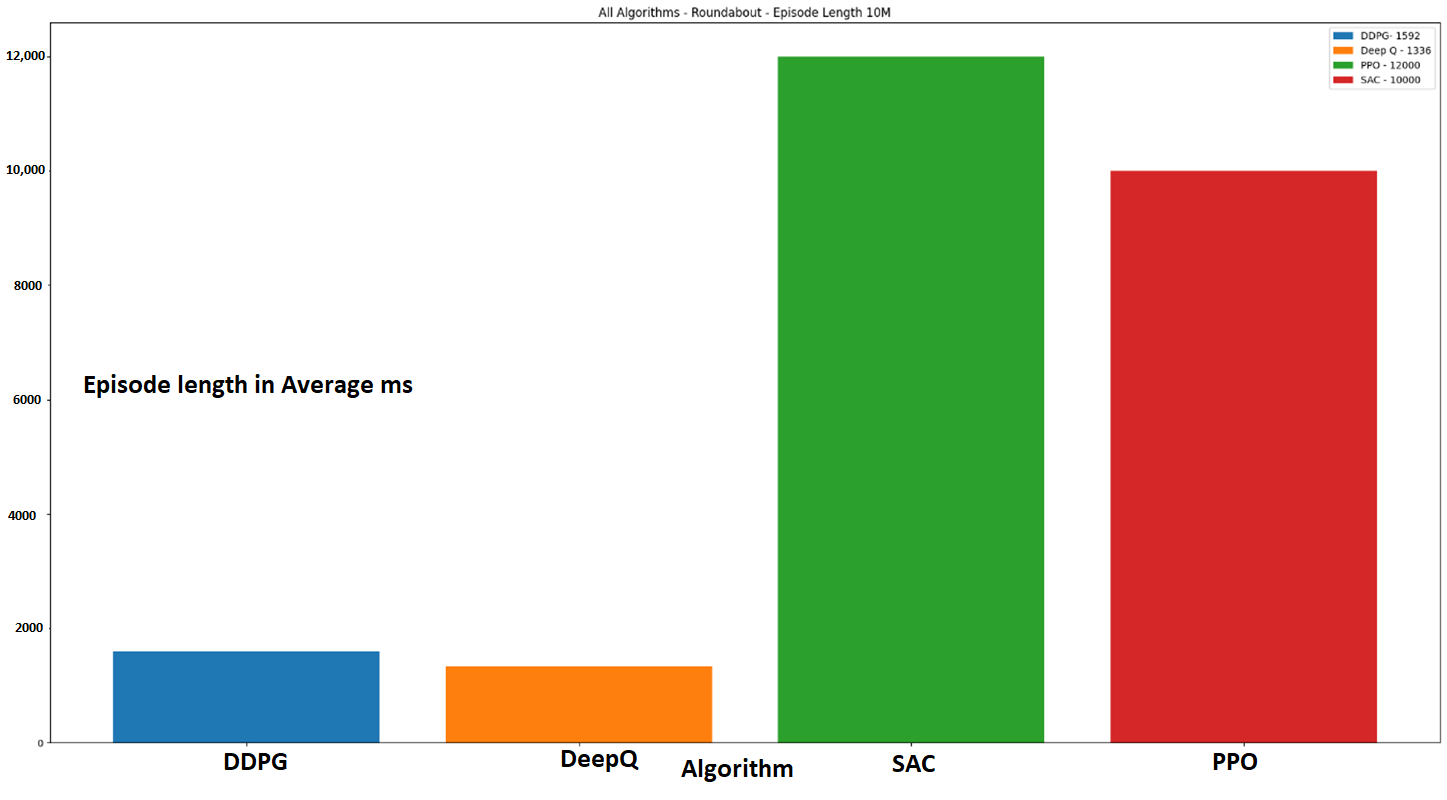

Supplement: Supplementary file 1 [file sensors-23-08649-s001.zip › images/Roundabout_EL.png]

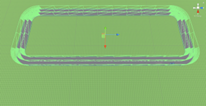

Supplement: Supplementary file 1 [file sensors-23-08649-s001.zip › images/Speeding.png]

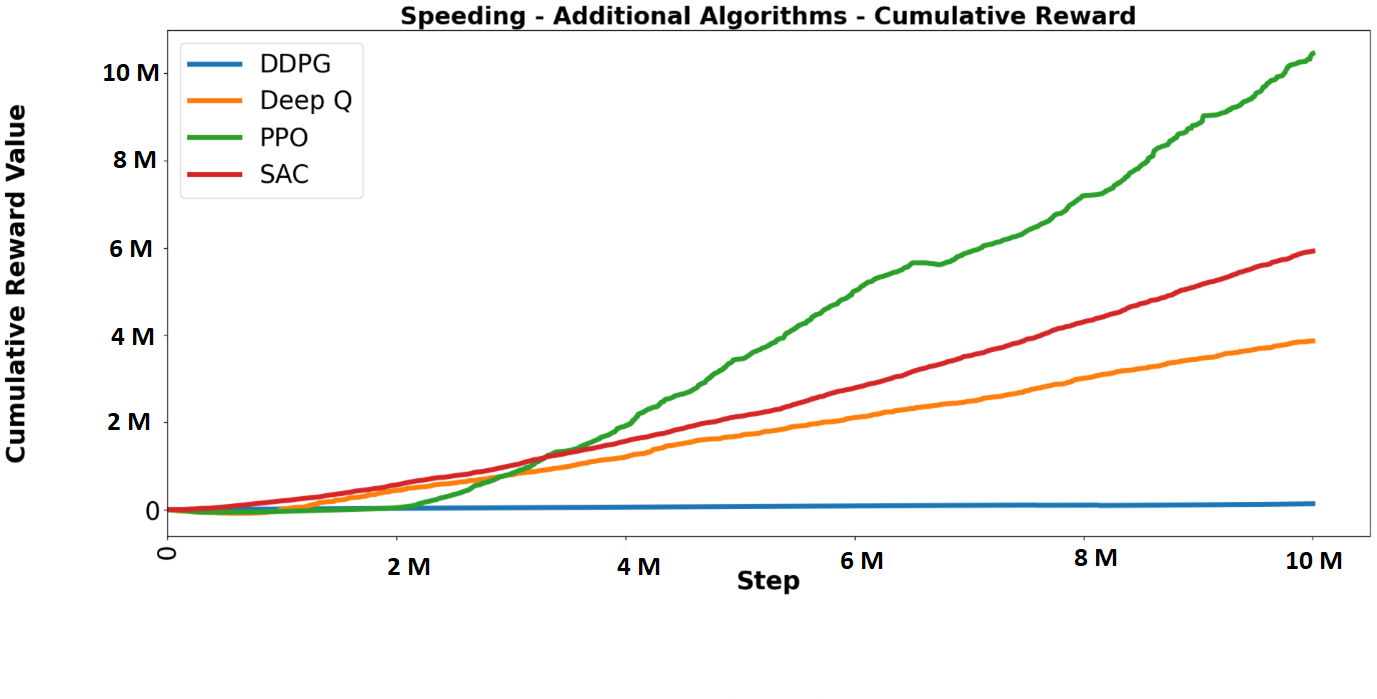

Supplement: Supplementary file 1 [file sensors-23-08649-s001.zip › images/Speeding_CL.png]

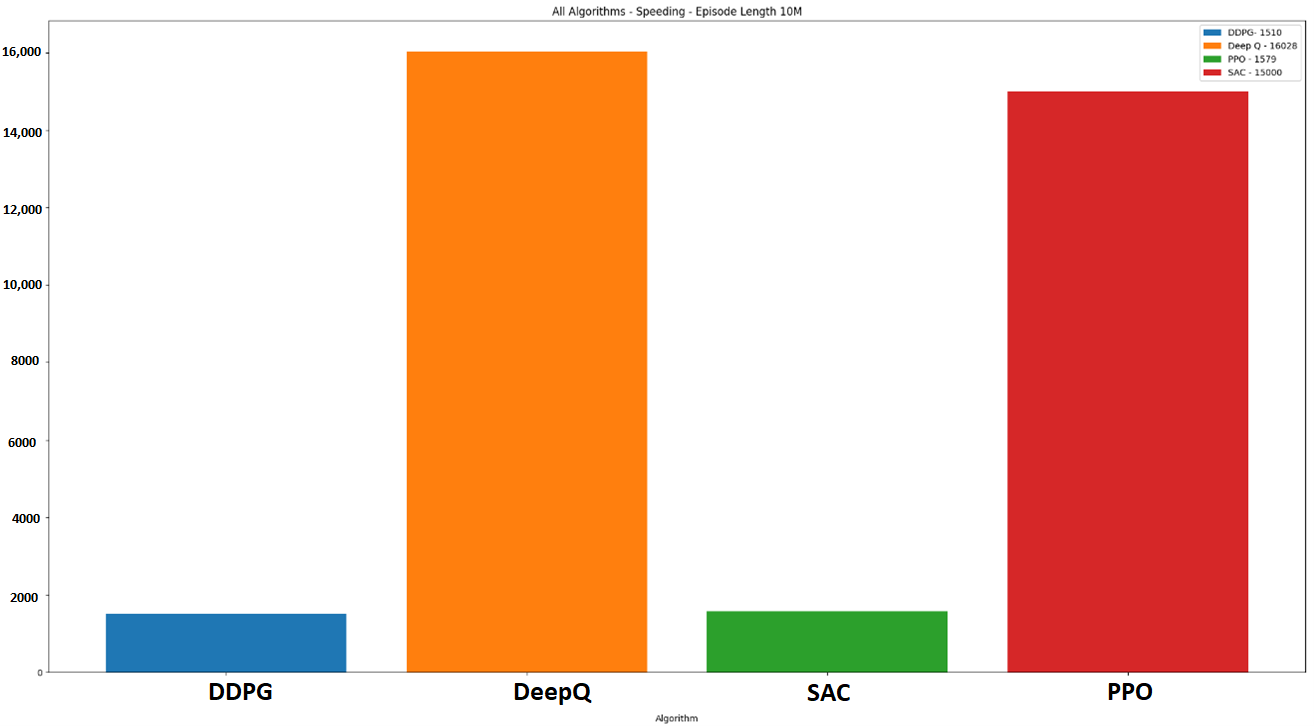

Supplement: Supplementary file 1 [file sensors-23-08649-s001.zip › images/Speeding_EL.png]
